# Inflammation and Skeletal Muscle Wasting During Cachexia

**DOI:** 10.3389/fphys.2020.597675

**Published:** 2020-11-19

**Authors:** Justine M. Webster, Laura J. A. P. Kempen, Rowan S. Hardy, Ramon C. J. Langen

**Affiliations:** ^1^Department of Respiratory Medicine, NUTRIM School of Nutrition and Translational Research in Metabolism, Faculty of Health, Medicine and Life Sciences, Maastricht University, Maastricht, Netherlands; ^2^Institute of Metabolism and Systems Research, University of Birmingham, Birmingham, United Kingdom; ^3^Centre for Endocrinology, Diabetes and Metabolism, Birmingham Health Partners, Birmingham, United Kingdom; ^4^Institute for Clinical Sciences, University of Birmingham, Birmingham, United Kingdom; ^5^MRC Arthritis Research UK Centre for Musculoskeletal Ageing Research, University of Birmingham, Birmingham, United Kingdom

**Keywords:** cachexia, inflammation, muscle wasting, atrophy, cancer, COPD, cytokines

## Abstract

Cachexia is the involuntary loss of muscle and adipose tissue that strongly affects mortality and treatment efficacy in patients with cancer or chronic inflammatory disease. Currently, no specific treatments or interventions are available for patients developing this disorder. Given the well-documented involvement of pro-inflammatory cytokines in muscle and fat metabolism in physiological responses and in the pathophysiology of chronic inflammatory disease and cancer, considerable interest has revolved around their role in mediating cachexia. This has been supported by association studies that report increased levels of pro-inflammatory cytokines such as tumor necrosis factor-alpha (TNF-α) and interleukin-6 (IL-6) in some, but not all, cancers and in chronic inflammatory diseases such as chronic obstructive pulmonary disease (COPD) and rheumatoid arthritis (RA). In addition, preclinical studies including animal disease models have provided a substantial body of evidence implicating a causal contribution of systemic inflammation to cachexia. The presence of inflammatory cytokines can affect skeletal muscle through several direct mechanisms, relying on activation of the corresponding receptor expressed by muscle, and resulting in inhibition of muscle protein synthesis (MPS), elevation of catabolic activity through the ubiquitin-proteasomal system (UPS) and autophagy, and impairment of myogenesis. Additionally, systemic inflammatory mediators indirectly contribute to muscle wasting through dysregulation of tissue and organ systems, including GCs via the hypothalamus-pituitary-adrenal (HPA) axis, the digestive system leading to anorexia-cachexia, and alterations in liver and adipocyte behavior, which subsequently impact on muscle. Finally, myokines secreted by skeletal muscle itself in response to inflammation have been implicated as autocrine and endocrine mediators of cachexia, as well as potential modulators of this debilitating condition. While inflammation has been shown to play a pivotal role in cachexia development, further understanding how these cytokines contribute to disease progression is required to reveal biomarkers or diagnostic tools to help identify at risk patients, or enable the design of targeted therapies to prevent or delay the progression of cachexia.

## Cachexia in Cancer and Chronic Disease

Cachexia is a complex metabolic syndrome resulting in severe weight loss, anorexia, and asthenia that occurs in many types of cancer and chronic inflammatory diseases (Evans et al., 2008). The nature of this weight loss is characterized by a pronounced systemic muscle wasting and weakness and includes marked loss of fat mass. Consequently, cachexia remains a significant co-morbidity in many diseases increasing mortality, reducing quality of life, and representing a major burden on healthcare providers. However, despite its impact in human disease the mechanisms that underpin cachexia remain poorly defined. In this review, we provide an overview and explore the latest insights into the contribution of systemic inflammation in cachexia, including both direct and indirect mechanisms of inflammatory muscle wasting.

Cachexia is highly prevalent in various types of cancers, with estimates of 30% in lung and head and neck cancer, and 41–45% in pancreatic and liver cancer, respectively (Anker et al., 2019). In addition, a high prevalence of cachexia has also been described for patients with chronic illnesses, including chronic heart failure (CHF; 10–39%) (Anker et al., 1997; Valentova et al., 2020), chronic kidney disease (CKD; 30–60%) (Mak and Cheung, 2006), chronic obstructive pulmonary disease (COPD; 5–33%) (Koehler et al., 2007; Sanders et al., 2016; Kwan et al., 2019), and rheumatoid arthritis (RA; 19–32%) (Santo et al., 2018) (although RA is often debated as being a cachectic disease as weight loss is not always a defining feature; Roubenoff, 2009). Cachexia is associated with decreased survival and quality of life in these conditions (Anker and Sharma, 2002; Reid et al., 2010; McDonald et al., 2019), and poor clinical outcome as is illustrated by increased postoperative mortality for cancer (Pausch et al., 2012) and CHF (Abel et al., 1976) and decreased response to radiation-, chemo-, and immunotherapy in the presence of cachexia (Sealy et al., 2020).

Diagnostic criteria for cachexia include weight loss in the presence of underlying illness of >5% in ≤12 months, or weight loss >2% in individuals with a low body-mass index (<20 kg/m^2^) or low muscle mass (Fearon et al., 2011), and the presence of decreased muscle strength, fatigue or anorexia, and abnormal biochemistry (Evans et al., 2008). Interestingly, the latter refers to the presence of increased inflammatory serum markers like interleukin-6 (IL-6) and C-reactive protein (CRP), suggesting inflammation as a shared characteristic of cachexia in these distinct conditions. Indeed, several clinical studies have reported elevated levels of inflammatory markers in cancer patients with cachexia. In non-small cell lung carcinoma, CRP, IL-6, and interleukin 8 (IL-8) serum levels were increased in cachectic compared to non-cachectic lung cancer patients (Op den Kamp et al., 2013). Riccardi et al. (2020) recently highlighted changes in plasma concentrations of markers of systemic inflammation in patients with cancer cachexia compared to weight-stable cancer patients. A positive correlation between pro-inflammatory cytokines and fatty acid lipid profile was reported in patients with gastrointestinal cancer cachexia, accompanied with augmented levels of CRP, and elevated levels of pro-inflammatory cytokines IL-6, tumor necrosis factor-alpha (TNF-α), and IL-8 (Riccardi et al., 2020). In patients with gastro-esophageal cancer, multiple regression analysis identified dietary intake and serum CRP concentrations as independent variables in determining the degree of weight loss, with a higher predicted effect than disease stage (Deans et al., 2009). Based on these and other studies, it is now recognized that a systemic inflammatory response is associated with weight and muscle loss and poorer outcomes in patients with cancer (Arends et al., 2017), and can be applied in identifying the various stages of cachexia (Douglas and McMillan, 2014). A recent systematic review by Abbass et al. (2019) revealed a consistent correlation between systemic inflammation and low skeletal muscle index determined through CT and DEXA scans in patients with various types of cancer, further implicating a link between inflammation and skeletal muscle mass loss in cancer cachexia.

Similarly, a correlation between cachexia and inflammation has also been described in chronic diseases. Cachectic patients with CHF often exhibit elevated levels of pro-inflammatory cytokines such as TNF-α, IL-6, interleukin-1 (IL-1), and interferon-γ (IFN-γ), in addition to glucocorticoids (GCs) (Anker et al., 1999). These molecules act as signaling ligands to directly and indirectly impact muscle and adipose tissue metabolism in favor of wasting (Jackman and Kandarian, 2004). Patients with cardiac cachexia also exhibit elevated levels of Angiotensin II (Ang II), which has been shown to increase the levels of these pro-inflammatory cytokines (Brink et al., 2001; Zhang et al., 2009), and induce cachexia (Brink et al., 2001). In CKD patients, increased expression of TNF-α and IL-6 is detected in skeletal muscle (Zhang et al., 2020), and cachexia is accompanied by increases in circulating TNF-α and IL-6 in addition to CRP (Stenvinkel et al., 2005), which often occur in the presence of malnutrition, and therefore is referred to as malnutrition-inflammation-cachexia syndrome (Kalantar-Zadeh, 2005). In patients with CKD, cachexia was not only associated with elevated levels of CRP, but also increased fibrinogen, and reduced cross-sectional area (CSA) of muscle fibers and fat mass (Zhang et al., 2013). Cachexia development in COPD has also been associated with the presence of systemic inflammation. COPD patients with unintentional weight loss and skeletal muscle mass loss showed elevated serum cytokine levels including TNF-α and IL-6 compared to stable-weight patients (Di Francia et al., 1994; Eid et al., 2001). Disease-specific characteristics of COPD have been implicated as the driver of inflammation, such as increased TNF-α levels as a result of hypoxemia resulting from reduced lung function (Takabatake et al., 2000), or increased systemic inflammation secondary to disease exacerbations in COPD (Wedzicha et al., 2000). Respiratory infections and subsequent pulmonary inflammation are a frequent cause of exacerbation, and these episodes are accompanied by catabolic changes in skeletal muscle (Crul et al., 2010), and considered as a potentially accelerating phase in COPD cachexia (Abdulai et al., 2018). It is well established that patients with RA have elevated levels of inflammatory cytokines, specifically TNF-α, IL-1β, and IL-6, and are found directly at the synovial joint or found to be released into the plasma, stimulating both local and systemic inflammation (Saxne et al., 1988; Shingu et al., 1993; Chikanza et al., 1995). The latter have been implicated in rheumatoid cachexia, with reports of reduced lean body mass (LBM) correlating with elevated serum levels of IL-6 and CRP (Munro and Capell, 1997; Engvall et al., 2008) In other chronic inflammatory conditions than RA, like Crohn’s disease, correlations with weight loss have also been described (Nakashima et al., 1998; Bossola et al., 2000; Mantovani et al., 2001; Dulger et al., 2004; Tas et al., 2005; Baker et al., 2016), indicating that the relation between systemic inflammation and cachexia may extend beyond the diseases described here. Importantly, a correlation between circulating inflammatory cytokines and cachexia may not be detected in all patients, as a result of the rapid systemic clearance, or the presence of additional triggers of muscle and weight loss in these complex conditions, such as malnutrition in CDK (Mak and Cheung, 2006) and COPD (Creutzberg et al., 2003), or hypoxemia in COPD (Ceelen et al., 2014). In addition, exacerbations in conditions like RA, COPD, and Crohn’s disease activity may be associated with transient increases in systemic inflammation, followed by sustained loss of body and muscle mass, which are not recovered as a result of other disease-related impairments in, e.g., inactivity, hypoxemia, or malnutrition (Ceelen et al., 2014).

The observed correlation between inflammation with cachexia across the various diseases described above is the basis for including serum markers of increased inflammation as a criterion in the definition of cachexia. Importantly, beyond its use as a clinical hallmark, inflammation has been investigated as a potential driver of cachexia, fueling a number of studies investigating the causal involvement of inflammation and underlying mechanisms by which it contributes to cachexia. An overview of these reports is presented below. To facilitate their discussion, a description of the processes that govern muscle mass is provided first.

### Cellular Processes That Determine Muscle Mass

Maintenance and modulation of skeletal muscle mass has been attributed to two processes: protein turnover and myonuclear turnover. Under normal conditions, these processes are maintained in homeostasis, however during skeletal muscle wasting the balance within these processes shift in favor of muscle wasting, through the inhibition of muscle protein synthesis (MPS), activation of muscle protein degradation, reduction in myonuclear accretion, or increased myonuclear loss. Protein turnover is a dynamic process determined by protein synthesis and degradation and is mediated through transcriptional, translational, and post-translational mechanisms (Booth et al., 1998). One important regulatory circuit of protein turnover is the insulin growth factor-1(IGF-1)–phosphoinositide−3−kinase (PI3K)–Akt/protein kinase B (PKB)–mammalian target of rapamycin (mTOR) pathway. Akt is the key mediator stimulating protein synthesis through mTOR activation, while inhibiting protein degradation through phosphorylation of transcription factor (TF) Forkhead box O (FoxO), leading to its cytoplasmic retention where it is inactive (Schiaffino et al., 2013). Translational capacity in skeletal muscle is regulated through eukaryotic initiation factors (eIFs) and ribosomal S6 kinase (P70S6K). Formation of the eIF4F complex is a rate-limiting step in initiation of the mRNA translation process (Gingras et al., 1999), while phosphorylation of P70S6K facilitates ribosomal biogenesis and translation (Hay and Sonenberg, 2004).

Skeletal muscle is the largest latent reservoir of amino acids, which are mobilized by increased proteolysis of mainly muscle contractile proteins to provide energy or precursors for protein synthesis to other vital organs (Attaix et al., 2005). Muscle protein degradation increases as a physiological response to starvation but is also activated during pathological catabolic states that accompany inflammation and cachexia. Control of muscle mass is highly regulated by proteolytic enzyme systems including the ubiquitin-proteasomal system (UPS), autophagy-lysosomal pathway (ALP), caspases, and calpains. The UPS is involved in the removal of specific proteins for degradation following marking with ubiquitin through specific a sequential process catalyzed by ubiquitin-activating enzymes (E1), conjugating enzymes (E2), and ligating enzymes (E3) (Sorensen, 2018). It is an enzymatic process initiated by E1 enzymes activating the ubiquitin which is transferred to the E2 ubiquitin-conjugating enzyme (Ciechanover, 2005). The E2 ubiquitin complex binds to E3 protein ligases that recognize substrate proteins that will be ubiquitinated. Polyubiquitinated proteins are transferred in an ATP-dependent manner to 26S proteasome complexes in which they are degraded. E3 Ub ligases are postulated as rate-limiting factors in this pathway (Scicchitano et al., 2018), and a number of muscle enriched E3 Ub-ligases have been described, including Atrogin-1 and MuRF1. The ALP is essential for removal of misfolded or aggregated proteins and damaged parts of the cell to prevent accumulation of toxic or abnormal organelles and proteins. In addition, it enables the breakdown of proteins to produce amino acids by skeletal muscle that can be utilized in other tissues during catabolic periods, such as starvation (Sandri, 2010). There are three types of autophagy described in mammals; macroautophagy, microautophagy, and chaperone-mediated autophagy. Although all three processes are distinct from one another, these mechanisms ultimately lead to lysosomal degradation of cargo and recycling of breakdown products (Bonaldo and Sandri, 2013). Calpains and caspases are families of cysteine proteases, and their proteolytic activity is increased during cellular necrosis or apoptosis. They have been implicated in suspending cell function through disabling signal transduction molecules by enzymatic cleavage at specific amino acid residues in a host of cells (Wang, 2000), but their overall relevance to increased muscle proteolysis in muscle atrophy is not yet clear. As will be highlighted in subsequent sections, both decreases in MPS and increases in proteolysis have been implicated in the loss of muscle mass in cachexia.

Myonuclear turnover is another important process involved in muscle homeostasis and is the balance between myonuclear accretion and apoptosis. Myonuclear accretion is the final step in post-natal myogenesis (Schiaffino et al., 2013), and relies on satellite cells (SCs), the local precursor cells of skeletal muscle (Pallafacchina et al., 2013). During muscle regeneration, SCs are activated and proliferate, which occurs through asymmetric cell division, resulting in two distinct myoblast populations. A portion of these myoblasts returns into quiescence to prevent depletion of precursor cells. The other population of myoblasts terminally differentiate and fuse with muscle fibers (or myotubes *in vitro*). Committed SCs highly express PAX7 and MYF5, which decreases during differentiation. MyoD is an important myogenic regulator during proliferation and early differentiation. Conversely, MyoG is most important in late differentiation, fusion and myotube formation (Bentzinger et al., 2012). Changes in the intricate regulation of SC activation, proliferation, and differentiation by triggers of muscle wasting, including inflammation, may result in impaired post-natal myogenesis, contributing to muscle atrophy. Myonuclear accretion seems to be impaired in cachexia as shown in animal studies (Ceelen et al., 2018a). In addition, in cachectic mice a reduced proliferation and differentiation capability of SCs was reported, resulting in myofibers not being able to regenerate or maintain their myofiber size by myonuclear accretion leading to atrophy (Inaba et al., 2018).

Evidence for a role of inflammation as a driver of cachexia and underlying mechanisms have mainly been collected in experimental models, including cultured skeletal muscle cells, animals exposed to controlled inflammatory conditions, and rodents in which diseases associated with cachexia are modeled. The marked benefit of these models is they can be deployed in experimental designs to address inflammation as a causative factor of cachexia. Moreover, they are instrumental to dissect direct and indirect effects of inflammation contributing to muscle atrophy, and provide fundamental insight in the underlying mechanisms, including the signaling pathways activated by inflammation and intra-cellular processes that cause muscle to atrophy. From these perspectives, the literature investigating the role of systemic inflammation in cachexia is discussed below.

### Associations Between Systemic Inflammation and Cachexia in Disease Models

Various experimental disease models have provided incremental associative evidence to imply inflammation in the etiology of muscle wasting. In murine cancer cachexia models, the concurrent presence of systemic inflammation and loss of muscle and fat tissue has extensively been document. In Walker-256 tumor-bearing rats increased systemic inflammation was observed with animals presenting elevated plasma TNF-α and IL-6 compared to non-tumor-bearing control animals (Cella et al., 2020). These also showed increased gene expression and protein levels of MuRF1 and Atrogin-1 in hind limb muscles (Cella et al., 2020). Similar observations were reported in an orthotopic mouse model of bladder cancer, in which increased levels of inflammatory cytokines TNF-α, IL-6, and IL-1β, and activation of pro-inflammatory pathways including NF-κB were detected in muscles of urothelial tumor-bearing animals. These observations were paralleled by downregulation of Akt- and FoxO3 phosphorylation levels, suggestive of a shift toward catabolic signaling (Chen et al., 2016). Zhuang et al. (2016) showed that Atrogin-1 and MuRF1 levels were significantly increased in colon-26 (C26)-bearing mice suffering from cachexia, along with significant increases in circulating and muscle TNF-α and IL-6, whereas expression levels of anabolic IGF-1 were decreased. A recent study by Chiappalupi et al. (2020) showed that body and muscle weight loss in mice with subcutaneously growing Lewis Lung Carcinoma (LLC) cells, was accompanied by elevated serum cytokine levels, including IL-1β, IL-6, IL-10, IFN-γ, and TNF-α. Interestingly, this study highlighted the importance of receptor for advanced glycation end-products (RAGE) in sustaining the inflammatory response in tumor-bearing mice, with RAGE null mice protected against increased systemic inflammation, body weight loss, and muscle weight loss (Chiappalupi et al., 2020).

The association between inflammation and cachexia has also been extensively explored in non-cancer, inflammatory models. Jepson et al. (1986) showed administration of LPS in fed rats resulted in inflammation and reduced muscle weights accompanied by decreased MPS and increased proteolysis of skeletal muscle. This study also showed fasted untreated animals had reduced MPS, yet fasting combined with LPS treatment exacerbated this reduction, therefore highlighting that reduced food intake alone in response to LPS was not the sole cause of skeletal muscle loss (Jepson et al., 1986). Therefore, these results show that the anorectic effects of LPS alone do not fully account for the muscle atrophy observed in this model, and therefore suggest anorexia-independent effects of inflammation in muscle wasting (Jepson et al., 1986). In line, Langen et al. (2012) showed animals with LPS-induced pulmonary and systemic inflammation exhibited more skeletal muscle atrophy than pair-fed control mice, implying an additional effect of inflammation beyond anorexia or hypophagia. Similarly, LPS administered to rats to induce sepsis resulted in reduced body weights and muscle weights which could not be fully attributed to hypophagia. Here, inflammation was implicated as the primary diver of skeletal muscle loss independently of anorexia (Macallan et al., 1996). Mice with LPS and pepsin aspiration pneumonia-induced inflammation showed increased levels of IL-1β, IL-6, and MCP-1 in diaphragm and limb muscles in combination with reduced myofiber size (Komatsu et al., 2018). In this model, increased levels of MuRF1 and Atrogin-1 in muscles were shown to be indicative of elevated UPS activity and calpain and caspase-3 pathway activation (Komatsu et al., 2018). Langen et al. (2012) showed pulmonary inflammation by intratracheal instillation of LPS in mice resulted in a rapid increase in circulating pro-inflammatory cytokines such as TNF-α, IL-1β, IL-6, and CXCL1, which preceded the loss of skeletal muscle mass in these animals. In both models of pulmonary inflammation, besides evidence for UPS-mediated proteolysis, increased Bnip3, LC3B, and Gabarapl1 expression levels were measured in skeletal muscle, suggesting elevated ALP activity in lung inflammation-induced muscle atrophy (Komatsu et al., 2018). Similar findings were reported in murine models of CHF. Here cardiomyopathy, characterized by increased pro-inflammatory macrophages infiltration in cardiac muscle and elevated serum IL-6 levels resulted in elevated skeletal muscle TNF-α and CXCR4 expression and reduced fiber CSA, indicative of inflammatory myopathy (Lynch et al., 2017; Song et al., 2019). Furthermore, CHF was shown to reduce skeletal muscle regeneration following muscle damage (Song et al., 2019).

Despite the diversity in primary pathology, and the degree and kinetics by which cachexia develops in these disease models, systemic inflammation and its preceding and correlation with muscle wasting appear a consistent factor beyond simple association in cachexia.

### (Pre)clinical Evidence for Inflammation as a Cause of Cachexia in Disease

Several *in vivo* studies have highlighted the importance of specific pro-inflammatory cytokines or activation of specific inflammatory pathways in muscle wasting through genetic or pharmaceutical inhibition, showing causal evidence that inflammation is a required component of atrophy in pathological models.

In models of cancer cachexia, e.g., mice or rats with methylcholanthrene-induced sarcoma (MCG-101) or in LLC tumor bearing mice, skeletal muscle wasting was alleviated through the blockade of the apex pro-inflammatory cytokine TNF-α through administration of anti-TNF antibodies (Sherry et al., 1989; Torelli et al., 1999). In addition, intra-muscularly inoculated LLC-tumor bearing WT mice displayed increased protein degradation and loss of muscle mass, which was prevented in transgenic littermates overexpressing the soluble TNF receptor type 1 protein (sTNF-R1) to inhibit the actions of local or circulating TNF-α (Llovera et al., 1998). While this study implies a causal contribution of TNF-α in skeletal muscle atrophy, this may result from indirect effects of TNF-α by increasing other cytokines such as IL-6 that contribute to cachexia development by impacting on skeletal muscle. Indeed, IL-6 has been implicated in cancer cachexia. Apc^*Min/+*^ mice with cancer cachexia showed significant muscle wasting in presence of a 10-fold increase of serum IL-6 levels compared to control groups (Baltgalvis et al., 2009). In addition, host-IL-6 was shown to be required in the development of cachexia in these mice, with Apc^*Min/+*^/IL-6^–/–^ mice showing reduced tumor burden and muscle wasting (Baltgalvis et al., 2008). Furthermore, administration of anti-murine IL−6 receptor antibody to C−26−bearing mice attenuated muscle wasting, in support of IL-6-dependent muscle atrophy in colon cancer cachexia (Fujita et al., 1996).

Administration of both anti-TNF-α and an IL1-receptor antagonists in models of cancer cachexia also shows evidence of preservation of body weight and improved food intake compared to untreated tumor-bearing controls, suggesting a common mechanism for both cytokines (Gelin et al., 1991). Furthermore, anti-TNF-α and anti-IL1-R treatment also reduced tumor growth, suggesting indirect effects of inflammation as a determinant of tumor burden in driving atrophy (Gelin et al., 1991). Similarly, in a rat RA model, administration of soluble TNF receptor I (sTNFRI) as a TNF blocking strategy improved body weight, but also food intake compared to control groups, implying that anti-inflammatory modulation may contribute to bodyweight maintenance by blocking anorexic effects (Granado et al., 2006). In line with that notion, in a cardiac cachexia rodent model, anti-TNF-α treatment significantly reduced losses in body and skeletal muscle mass, partly through reduced UPS activation through the attenuation of anorexia (Steffen et al., 2008). Finally, in a model of cachexia induced by *Trypanosoma cruzi*, mice treated with anti-TNF-α antibodies displayed significant attenuation of weight loss, while anti-IL-6 and anti-IFN-γ antibodies had no such effect. In addition, this protection of weight loss occurred during the acute phases of infection and was only transient in nature, suggesting early administration of anti-TNF therapies may be more effective in the early phases of cachexia (Truyens et al., 1995).

The concept of a causal role of pro-inflammatory cytokines mediating cachexia in humans has only been addressed very limitedly and only in a few pathological conditions, using targeted therapeutics that deplete specific cytokines such as TNF-α and IL-6. In contrast to the experimental models, the effects of TNF-α blockade in cancer cachexia, while complicated by the diversity of disease etiologies, and limitations due to the actions of the corresponding treatments on tumor immunity, have proven less promising. Here a number of studies examining different cancer patient cohorts have failed to identify any meaningful changes in body weights, LBM, or muscular function (determined by 6 min walk test) in response to anti-TNF-α interventions (Jatoi et al., 2007, 2010; Wiedenmann et al., 2008; Gueta et al., 2010). Studies examining anti-IL-6 in cachexia are limited. One case report in a patient with large-cell carcinoma of the lung and cancer cachexia demonstrated improved inflammatory outcomes with reduced serum IL-6 after prednisolone treatment, with no further deterioration in cachexia parameters (Ando et al., 2013). While these studies are frequently complicated by poor accrual, recruitment, and interactions with other disease-related complications and between ongoing therapeutic interventions, thus far the causal involvement of inflammation in muscle atrophy during cancer cachexia has been difficult to assess in clinical studies.

In COPD, several TNF blocking agents have reached phase-II clinical trials, but these have been complicated by initial concerns related to increased incidence of cancer compared to the placebo control treatment arms (Rennard et al., 2007), which were later contested in a long-term follow-up analysis (Rennard et al., 2013). While the original rationale for anti-TNF treatment was to intervene in the lung pathology, more recent insights suggest that specific groups of patients, in particular COPD patients with cachexia may benefit from TNF-blocking agents (Rennard et al., 2013). Studies appropriately designed to assess this remain to be initiated, however. In contrast, TNF-α blockade has proven highly effective in the management of chronic inflammatory disease such as RA (Saxne et al., 1988). It must be noted that in RA, evaluating the direct contribution of inflammation on muscle mass and function is complicated by the actions of these treatments on disease activity, which reduce pain and allow for improvements in dietary intake and physical activity. Here, several such studies failed to report meaningful correlations in changes in body composition, in response to anti-TNF-α treatments over short durations (Marcora et al., 2006; Elkan et al., 2009). These studies are complicated by side by side comparison with disease modifying anti-rheumatic drugs such as methotrexate, which possess their own anti-inflammatory immunomodulatory effects that may mask muscle protective actions. However, studies examining anti-TNF-α intervention in RA over longer periods revealed improvement in body weight, BMI, total and fat mass relative to patients receiving standard disease management treatment (Chen et al., 2013; Toussirot et al., 2014). Moreover, promising results have been observed in alternative chronic inflammatory arthropathies and inflammatory diseases such as ankylosing spondylitis and Crohn’s disease. Here, in ankylosing spondylitis, improvement in muscle strength parameters was reported following anti-TNF-α intervention, while increases in both muscle volume and strength were evident in patients with Crohn’s (Subramaniam et al., 2015; Demirkapi et al., 2017).

Despite the ambiguous evidence from clinical studies, the preclinical disease models strongly support a causal relationship between inflammation and cachexia. The extent to which actions of inflammation depend on interactions with other pathology-related alterations, or whether inflammation *per se* is sufficient to induce cachexia, is addressed in different experimental models described next.

### Causal Evidence Implying Inflammation as a Driver of Skeletal Muscle Atrophy

Many *in vivo* studies have shown that the induction of an inflammatory state by TNF-α infusion initiate the development of cachexia, resulting in reduced food intake, loss of bodyweight, and skeletal muscle loss (Tracey et al., 1988; Llovera et al., 1993). In addition, implantation of a continuously TNF-α producing tumor cell line into mice, elicited cachexia and weight loss, with reduced food intake, compared to the control, non-secreting tumor cell line (Oliff et al., 1987). TNF-α has also shown to suppress the IGF-1 pathway and cause insulin resistance, which may also play a role in the dysregulation of macronutrient uptake and utilization (Broussard et al., 2003; Frost et al., 2003). Mice inoculated with tumors that overexpress IFN-γ presented with severe cachexia, and IFN-γ inhibition prior to inoculation attenuated body weight loss (Matthys et al., 1991), implying IFN-γ secretion rather than other tumor-dependent effects in the development of tissue depletion in this model. In mice inoculated with tumor cells expressing Fn14, a receptor for the inflammatory cytokine tumor necrosis factor-like weak inducer of apoptosis (TWEAK), significant cachexia development and reduced survival rates were observed (Johnston et al., 2015). Moreover, anti-Fn14 monoclonal antibodies prevented cachexia development, while tumor growth rate being reduced, implying local or reciprocal effects of anti-Fn14 on tumor growth and cachexia (Johnston et al., 2015). In line, chronic administration or muscle-specific transgenic overexpression of TWEAK in mice resulted in reduced body and skeletal muscle weight with an associated increased activity of UPS and NF-κB (Dogra et al., 2007).

Alternatively, the release of pro-inflammatory cytokines is triggered in models of sepsis. Schakman et al. (2012) induced inflammation in rats through LPS injection, which lead to a loss of body and muscle weight. IGF-1 levels were significantly reduced, and accompanied by an upregulation of FoxO1, Atrogin-1, and MuRF1. In a similar model, identical findings were reported, as well as increased muscle TNF-α expression (Dehoux et al., 2003), indicative of local inflammatory signaling and activation of proteolysis in this model of muscle wasting. Increased levels of systemic inflammatory cytokines may result from spill over from an inflamed site, as is the cause for pulmonary inflammation. Ceelen et al. (2018b) evoked pulmonary inflammation in mice, which resulted in systemic inflammation and muscle atrophy, with accompanying of UPS-mediated proteolysis and upregulation of E3 ligases MuRF1 and Atrogin-1. Interestingly, there were no additive effects of body weight loss and muscle wasting in emphysematous mice compared to control groups after LPS exposure, and can therefore be concluded muscle atrophy was a direct consequence of the pulmonary inflammation and not affected by the presence of emphysema (Ceelen et al., 2017). This group also showed evoking repetitive pulmonary inflammation in emphysematous mice, mimicking recurrent acute exacerbations in COPD, resulted in sustained muscle atrophy, which was associated with markers of impaired muscle regeneration, with altered myogenic signaling and reduced fusion capacity (Ceelen et al., 2018a). In line, chronic pulmonary inflammation in transgenic mice with lung-specific overexpression of TNF resulted in muscle atrophy and an impaired muscle regenerative response compared to WT littermate control animals (Langen et al., 2006).

Taken together, these studies demonstrate inflammation is sufficient to drive muscle wasting beyond the context of disease-induced cachexia. However, these models cannot distinguish direct from indirect effects, i.e., requiring involvement of another tissue or intermediary paracrine or autocrine signal, impacting on the intramuscular processes that drive muscle mass loss in cachexia. As such understanding is instrumental for development of intervention strategies, an extensive number of studies focused on identifying the intracellular pathways responsible for sensing inflammatory signals and transducing these into atrophy responses.

## Direct Effects of Inflammation: Signaling Pathways and Activating Ligands Responsible for Relaying Direct, Muscle Atrophy-Inducing Effects of Inflammation

**FIGURE 1:**
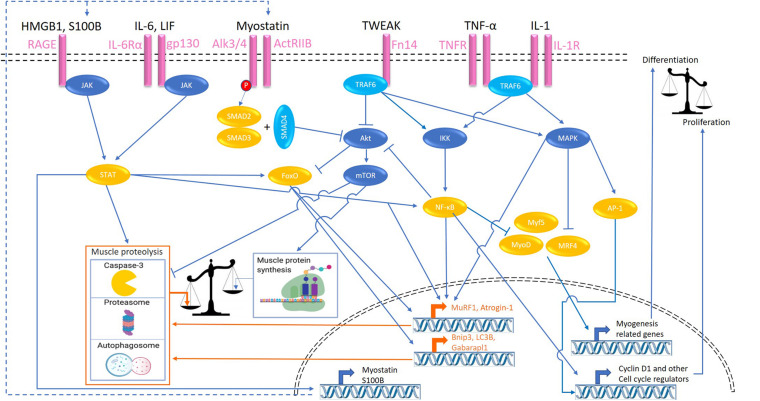
Signaling pathways activated by inflammatory ligands involved in cachexia-related muscle atrophy. Colors refer to transcription factors (orange), proteolytic signaling (orange), kinases (dark blue), adaptor proteins (light blue), and cell surface receptors (pink). HMGB1, high mobility group box 1; S100B, S100 calcium-binding protein B; RAGE, receptor for advanced glycation endproducts; ActRIIB, activin receptor type IIB; TWEAK, tumor necrosis factor-like weak inducer of apoptosis; Fn14, fibroblast growth factor-inducible 14; TNFα, tumor necrosis factor-α; IL, interleukin; JAK, Janus kinase; STAT, signal transducers and activators of transcription; TRAF, TNF receptor associated factor; FoxO, Forkhead box transcription factors; mTOR, mammalian target of rapamycin; IKK, IκB kinase; NF-κB, nuclear factor-κB; MAPK, mitogen-activated protein kinase; JNK, c-Jun N-terminal kinase; MyoD, myoblast determination protein 1; MyoG, myogenin; MRF4, myogenic regulatory factor; AP-1, activator protein 1; MuRF-1, muscle RING-finger protein-1; Bnip3, BCL2 interacting protein 3; Gabarapl1, GABA type A receptor associated protein like 1.

The direct effects of inflammation on skeletal muscle require receptor-mediated activation of intra-muscular signaling pathways. Various signaling pathways activated by inflammatory cytokines, or inflammation-associated ligands have been implicated in muscle atrophy through regulation of muscle protein turnover or myonuclear turnover. These pathways and the corresponding activating ligands (overview in Figure 1) that have been implicated in muscle atrophy are described in this section, including their impact on muscle protein and myonuclear turnover.

### NF-*κ*B-Signaling

Nuclear factor-κB (NF-κB) is a TF, and activation of classical NF-κB signaling occurs in response to various inflammatory cytokines (e.g., IL-1 and TNF-α) and oxidative stress. The former act through corresponding receptor binding and activation, and recruitment of adaptor proteins, resulting in I-kappa-B kinase (IKK) activation and culminating in NF-κB nuclear translocation. TNF receptor-associated factors (TRAF) is a family of intracellular adaptor proteins that interact with the surface receptors TNFR-1 and -2, Toll-like receptor 4 (TLR4), and IL-1R. TNF-α receptor adaptor protein 6 (TRAF6) not only integrates upstream inflammatory signals, but is central to the activation of many signaling pathways including NF-κB and MAPK in response to cytokines (Paul et al., 2010; Miao et al., 2017). Activity if this adaptor protein is elevated in cachectic LLC-bearing mice, while TRAF6 depletion attenuated muscle wasting in tumor bearing mice (Paul et al., 2010). Downstream, NF-κB activation has been implicated as an important step in inflammation-induced muscle wasting. Inhibition of NF−κB alleviates the cytokine-driven atrophy of muscle, thus highlighting NF-κB in the direct effects of inflammatory stimuli in muscle wasting (Ladner et al., 2003). Prevention of muscle NF-κB activation in genetically modified mice attenuated muscle wasting in a model of pulmonary inflammation-induced systemic inflammation (Langen et al., 2012). Similarly, both, pharmacological (Miao et al., 2017) and muscle-specific genetic (Cai et al., 2004) inhibition of NF-κB prevented muscle wasting in tumor bearing mice, indicating the importance of this TF in cancer cachexia. Conversely, muscle specific activation of the NF−κB pathway in transgenic mice resulted in profound muscle atrophy (Cai et al., 2004).

NF-κB activation has been implicated in increasing UPS proteolytic activity through the expression of E3 Ub-ligases genes Atrogin-1 and MuRF1 (Kandarian and Jackman, 2005; Dillon et al., 2007; Schiaffino et al., 2013). In lung inflammation-induced muscle atrophy, genetic inhibition of skeletal NF−κB inhibited the increases in MuRF1 expression (Langen et al., 2012), which was shown as a required step for muscle wasting in a similar model using MuRF1 knock-out (KO) mice (Files et al., 2012). In line, upregulation of MuRF1 was also required for muscle atrophy observed in response to muscle specific activation of the NF−κB pathway in transgenic mice (Cai et al., 2004). Furthermore, NF-κB, through Akt inhibition, leads to elevated FoxO activity which stimulates the expression of UPS- and ALP-related genes, such as LC3 and Bnip3 (Hanna et al., 2012). Additionally, NF-κB prevents myoblast cell cycle exit, reduces MyoD and Myf-5 protein abundance and activity (Langen et al., 2004), and decreases MyoD mRNA expression (Guttridge et al., 2000), leading to impaired post-natal myogenesis (Guttridge et al., 2000; Langen et al., 2001, 2004). Furthermore, *in vitro* and *in vivo* studies have also shown that serum factors from cachectic mice and patients, in an NF-κB-dependent manner, induce expression of the self-renewing factor Pax7, implying NF-kB inhibits myogenic differentiation through sustained Pax7 expression (He et al., 2013).

Muscle wasting-inducing properties of the inflammatory cytokines TNF-α and IL-1 have mainly been attributed to receptor-mediated activation of NF-κB, and involve increased proteolysis as well as impaired myogenesis, Several *in vitro* studies have shown inflammatory cytokine TNF-α administration in C2C12 myocytes leads to activation of NF−κB signaling (Guttridge et al., 2000; Langen et al., 2001). The NF−κB inhibitor PDTC inhibited upregulation of MuRF1 induced by TNF-α medium *in vitro* through inhibition of NF−κB indicating the importance of this signaling pathway. TNF-α induced upregulation of the catabolic genes Atrogin-1 and MuRF1 parallel to inducing myotube atrophy in differentiated C2C12 cells (Li et al., 2005). In L6 and C2C12 myotubes treated with TNF-α, decreased eIF3f translation initiation factor abundance and increased Atrogin-1 levels were observed during myotube atrophy, suggestive of decreased protein synthesis and elevated proteolysis, respectively (De Larichaudy et al., 2012). Genetic inhibition of NF-κB was also reported to prevent TNF-induced myotube atrophy (Liu et al., 2010), although in later work by this group TNF-α was postulated to act via p38 to increase Atrogin-1 and MuRF1 (Li et al., 2005), which was confirmed in another study for TNF-α-induced Atrogin-1 in C2C12 myotubes (Chiappalupi et al., 2020). IL-1 is an inflammatory cytokine which actions overlap with TNF-α, and can be elevated during cancer cachexia (Cederholm et al., 1997). C2C12 incubation with either IL-1α or IL-1β resulted in reduced myotube size and activation of NF-κB signaling, in turn leading to increased Atrogin-1 and MuRF1 expression (Li et al., 2009). Another activator of NF-kB signaling implicated in muscle atrophy concerns the cytokine TWEAK and its receptor fibroblast growth factor inducible 14 (Fn14). TWEAK has been shown to be capable of inducing inflammation, which was reduced in Fn14-deficient mice, through unknown mechanisms, implying TWEAK as a feed-forward signal for an inflammatory state (Girgenrath et al., 2006). Following TWEAK binding to Fn14 it can activate various signaling modules through its adaptor proteins (e.g., TRAF6), leading to NF-κB and MAPK activation (Sato et al., 2014). Myotubes incubated with TWEAK show increased NF-κB activation (Dogra et al., 2007; Bhatnagar and Kumar, 2012), MuRF1 and ALP-related genes such as Beclin1, and activation of caspases. Inhibition of MuRF1, autophagy, or caspase-3 blocked the TWEAK-induced degradation of MyHC and myotube atrophy (Bhatnagar et al., 2012). Furthermore, TWEAK incubation *in vitro* can inhibit Akt phosphorylation, leading to reduced protein synthesis while stimulating protein degradation (Dogra et al., 2007).

*In vivo*, TNF-α overexpression has been shown to impair proliferative and myogenic responses during muscle regeneration (Langen et al., 2006). In differentiating C2C12 myocytes, activation of NF-κB by TNF-α incubation lead to the inhibition of MyoD through destabilization of MyoD mRNA (Guttridge et al., 2000) and MyoD protein (Langen et al., 2004). Another study showed that when using the NF−κB inhibitor PDTC the induction of atrogenes which may have contributed to MyoD and MyHC proteolysis was inhibited in cells incubated with TNF-α (Miao et al., 2017). TNF-α has been shown to stimulate myoblast proliferation at the expense of differentiation *in vitro* in an NF-κB-dependent manner (Otis et al., 2014). Similarly, it was also shown IL-1 induces proliferation which was inhibited after inhibiting NF-κB, indicating this effect is NF-κB mediated (Otis et al., 2014). Two other studies also showed IL-1 stimulated NF-κB activity *in vitro*, which also showed increases in proliferation of both primary and C2C12 myoblasts, highlighting IL-1 impacts on myogenic activity in skeletal muscle cells (Langen et al., 2001; Otis et al., 2014). TWEAK has also been shown to convey anti-differentiation and pro-proliferation actions through inducing sustained NF-κB activation and MyoD degradation in addition to reduced expression levels of MyoD and MyoG *in vitro* (Dogra et al., 2006; Girgenrath et al., 2006; Winkles, 2008).

Besides direct atrophy-inducing effects of TNF signaling through NF-kB, autocrine activation of parallel pathways in muscle have also been described. Treatment of TNF-α alone or combined with IFN-γ increased the expression of RAGE and its ligands S100B, and HMGB1 in C2C12 myotubes (Chiappalupi et al., 2020). Subsequent atrophy of myotubes and increased Atrogin-1 and MuRF1 mRNA expression levels required the presence of RAGE, and involved JAK-STAT activation, implying an autocrine signaling circuit downstream of TNF-induced p38 MAPK and NF-κB activity.

### JAK/STAT-Signaling

The JAK/STAT pathway is activated by type I (IFN-α/β), type II (IFN-γ), IL-2, and IL-6 receptor stimulation (Schindler et al., 2007). IL-6 binding to the IL-6r-Gp130 receptor complex results in the recruitment to the intracellular domain of the receptor, and subsequent activation of the JAK tyrosine kinase. After binding, JAK proteins undergo a conformational change, dimerize, and activate the STAT proteins through phosphorylation. Subsequently homo- or hetero-dimerization of STAT proteins is followed by translocation to the nucleus (Moresi et al., 2019). STAT transcriptional activation contributes to muscle wasting through various mechanisms. It stimulates CCAAT/enhancer binding protein (C/EBPδ) expression and activity, which in turn increases myostatin, MAFbx/Atrogin-1, MuRF1, and caspase-3 expression in myofibers (Haddad et al., 2005; Zhang et al., 2013; Silva et al., 2015), enhancing muscle proteolysis. Moreover, increased myostatin expression resulting from STAT-C/EBPδ activation suppresses post-natal myogenesis (Zhang et al., 2013), which in turn may negatively affect muscle mass maintenance. Furthermore, STAT was documented to regulate gene transcription by interaction with FoxO and NF-κB (Oh et al., 2012; Yoon et al., 2012).

Interleukin-6 is a pleiotropic cytokine which can induce several intra-cellular signaling pathways including JAK/STAT in a variety of cells types. Intra-cellular signaling through the binding of IL-6 to the IL-6R in turn associates with the transmembrane protein Gp130, which is ubiquitously expressed in most cells. The soluble form of IL-6R (sIL-6R) is found in most bodily fluids and also binds to IL-6, further increasing the range of target tissues for IL-6 as the IL-6-sIL-6R complex has the ability to bind and activate to Gp130 on any cell, this is known as “trans- signaling” (Heinrich et al., 2003). IL-6 has been implicated as a core mediator of cancer cachexia. Indeed, systemic IL-6 concentrations increase with intestinal tumor development in Apc^*Min/+*^ mice and is associated with elevated p-STAT-3 and Atrogin-1 mRNA levels (Baltgalvis et al., 2009). In addition, host-IL-6 was shown to be required in the development of cachexia in these mice, with Apc^*Min/+*^/IL-6^–/–^ mice showing reduced tumor burden and muscle wasting (Baltgalvis et al., 2008). In line, increased circulating IL-6 levels and elevated STAT-3 signaling were detected in skeletal muscle of C26 cachectic mice, and inhibition of STAT-3 attenuated muscle atrophy *in vitro* and *in vivo* (Bonetto et al., 2012). Blockade of IL-6R through administration of an anti-murine IL-6R antibody in C26-bearing mice also showed attenuated muscle loss and reduced expression of cathepsin B and L in muscle compared to tumor-bearing controls (Fujita et al., 1996), highlighting the requirement of IL-6 and IL-6 signaling in this experimental model of cancer cachexia. Conversely, overexpression of human IL-6 increased expression of proteasomal subunits cathepsins B and L in muscle and induced muscle atrophy in transgenic mice (Tsujinaka et al., 1995), highlighting that chronic elevation of circulating IL-6 is sufficient to cause muscle wasting. In support of a role for increased proteolysis, intra-peritoneal injections of IL-6 lead to increased muscle atrophy in rats measured by tyrosine and 3-methylhistidine release (Goodman, 1994). In addition, reduced phosphorylation of PS6K1, indicative of reduced translational capacity, and protein synthesis have been reported in muscles infused with IL-6 (Haddad et al., 2005). Local IL-6 infusion into the TA muscle decreased total and myofibrillar protein content in rats (Haddad et al., 2005), suggesting that atrophy-inducing effects of IL-6 are the result of direct actions of IL-6 on skeletal muscle. In support of this, C2C12 myotubes treated with recombinant IL-6 did show reduced myotube diameter, reduced mTOR and 4EBP-1 phosphorylation, and increased STAT3 phosphorylation and Atrogin-1 transcription, showing IL-6 suppresses mTOR and therefore reducing protein synthesis, in addition to increasing atrogene expression (White et al., 2013). However, other studies have shown little effect of IL-6 on skeletal muscle both *in vivo* and *in vitro* (García-Martínez et al., 1994; Ebisui et al., 1995), which may be explained by differences in IL-6 levels, shorter exposure regiments, or the pleiotropic nature of IL-6 in skeletal muscle, e.g., the source of IL-6 (Daou, 2020).

Gp130 has been implicated as the main cellular receptor in skeletal muscle to mediate the IL-6 effects in cancer cachexia. Mice injected with LLC with a genetic deletion of Gp130 specifically in skeletal muscle showed attenuated muscle wasting compared to WT controls, primarily through reduced STAT signaling and atrogin-1 and FoxO3 activation (Puppa et al., 2014). In line, hyperactivation of STAT3 signaling through Gp130 activation in gp130^*F/F*^ knock-in mice with a k-Ras-driven lung carcinoma developed cachexia with reduced muscle and fat mass and reduced life expectancy compared to k-Ras mice without Gp130 hyperactivation (Miller et al., 2017). Although these results show the importance of the activation of Gp130 and STAT signaling in cancer cachexia, Gp130 activation is not solely restricted to IL-6.

Leukemia inhibitory factor (LIF) has also recently been identified as a cytokine, which activates the same receptor as IL-6 and also mediates skeletal muscle atrophy through STAT and ERK signaling (Seto et al., 2015). Seto et al. showed in a murine model of C26 colon carcinoma with muscle atrophy, serum levels of LIF increased in parallel to tumor development. LIF was actively secreted by C26 tumor cells, whereas TNF-α and IL-6 were not, and incubation of C2C12 myotubes with LIF was sufficient to induce atrophy. Conversely, LIF inhibition in tumor cell conditioned media (CM) prevented CM-induced myotube atrophy *in vitro*, while genetic inactivation of STAT3 in myofibers was sufficient to suppress atrophy *in vivo* (Seto et al., 2015). RAGE is part of the immunoglobulin superfamily and known as a key mediator of several pathological processes. It is activated by ligands including high mobility group box 1 (HMGB1) and the S100 calcium−binding protein B (S100B), which are secreted by various cell types, including tumor cells and damaged myofibers (Chiappalupi et al., 2020). Furthermore, inflammatory cytokines activate a feed-forward RAGE signaling loop by inducing HMGB1, S100B, and RAGE expression in skeletal muscles (Chiappalupi et al., 2020). Its potential relevance to cancer cachexia is suggested by increased serum levels of S100B and HMGB1 in the serum of cancer patients (Miyamoto et al., 2016; Chiappalupi et al., 2020), and highlighted by the observation that LLC-bearing RAGE/KO mice displayed delayed body and muscle weight loss, reduced Atrogin-1 and MuRF1 expression levels, and prolonged survival time compared to WT mice. The cytosolic domain of RAGE connects to JAK/STAT3 signaling, implicated in increased protein degradation and decreased differentiation, but has also been reported to activate the tyrosine kinase protein, Src, which is implicated in several other downstream signaling hubs, such as ERK1/2, p38 MAPK, JNK, and NF-κB (Riuzzi et al., 2018).

### MAPK Signaling

The MAPK pathway controls growth and stress responses in a myriad of cell types, including skeletal muscle. MAPK signaling is activated by cellular stress, growth factors, and pro-inflammatory cytokines (e.g., IL-1 and TNF-α) (Zhang and Liu, 2002). The MAPK family of proteins consists of four distinct signaling pathways, namely, extracellular signal-regulated kinases 1 and 2 (ERK1/2), p38 MAPK, c-Jun NH2-terminal kinases (JNK), and ERK5 (Kramer and Goodyear, 2007). p38 MAPK mediates upregulation of MuRF1 and Atrogin-1 in response to TNF-α by an unknown mechanism (Li et al., 2005; Chiappalupi et al., 2020). IL-1 signaling has also been shown to stimulate phosphorylation of p38 MAPK, leading to increased atrogin-1 expression, independent of Akt/FoxO signaling (Li et al., 2009). Furthermore, p38 phosphorylates MRF4, thus inhibiting the expression of selective myogenic genes in late myogenesis, and antagonizes the JNK proliferation-promoting pathway (Suelves et al., 2004; Perdiguero et al., 2007). JNK mediates AP-1 activation, which is a signaling molecule that controls proliferation and differentiation through transcriptional regulation of cell-cycle regulators such as cyclin D1, cyclin A, and cyclin E (Kyaw et al., 2002; Hess et al., 2004), and has been implicated in muscle atrophy responses (Liu et al., 2010). When treated with TNF-α, C2C12 increased p-ERK in differentiating myoblasts, which correlated with suppressed MyoD and MyoG levels, and reduced accretion of myosin heavy chain content. Administration of the ERK inhibitor PD98059 to C2C12 cells prevented this inhibitory effect of TNF-α on myogenic differentiation (Penna et al., 2010).

### SMAD-Signaling

The smad pathway is activated by multiple ligands, but in the context of muscle mass control, Myostatin/GDF8, a member of the transforming growth factor-β (TGF-β) family, is the best described (Lee and Jun, 2019), next to GDF11 (Egerman et al., 2015) and Activin-A (Trendelenburg et al., 2012). Myostatin is a myokine, and its autocrine and paracrine effects act as a break on skeletal muscle growth. Myostatin has been found to be associated with cancer cachexia and its expression is stimulated through the JAK/STAT pathway (Costelli et al., 2008; Zhang et al., 2013). This positions Smad signaling secondary to transcriptional activation of Mstn by inflammatory cues. Binding of myostatin to ActRIIB results in the phosphorylation of Smad2/3 (El Shafey et al., 2016) and activation of Smad signaling, which reduces p-Akt levels (Trendelenburg et al., 2009), consequently activating caspase-3 and FoxO, and resulting in increased protein degradation (Schiaffino et al., 2013; Zhang et al., 2013). Accordingly, downregulation of p-Akt and p-FoxO3 accompanied by myostatin and activin A overproduction in the muscle were seen in mice with bladder cancer (Chen et al., 2016), implying myostatin involvement in cachexia triggered by various cancer types (Zimmers et al., 2002; Chen et al., 2016). Myostatin administration is sufficient to induce cachexia in mice through ActRIIB signaling (Costelli et al., 2008). Conversely, blockade of the ActRIIB receptor prevented cachexia in C26 tumor bearing mice, without affecting increased circulating levels of IL-6, TNF-α, and IL-1β (Zhou et al., 2010), implying ActRIIB signaling acts independent, or downstream of inflammation-associated muscle atrophy through autocrine expression of ActRIIB activating ligands like myostatin or Activin-A (Trendelenburg et al., 2012). In support of this notion, inhibition of myoblast differentiation by inflammatory cytokines was found to require *de novo* Activin-A production (Trendelenburg et al., 2012), implying smad signaling secondary to an autocrine mechanism activated by inflammation.

Combined, these studies identify a myriad of inflammatory cytokines and ligands as mediators of inflammation, which directly impact on skeletal muscle through receptor-mediated signaling which affects muscle protein turnover in favor of proteolysis or impairs myogenesis, ultimately resulting in muscle wasting.

## Indirect Effects of Inflammation

**FIGURE 2:**
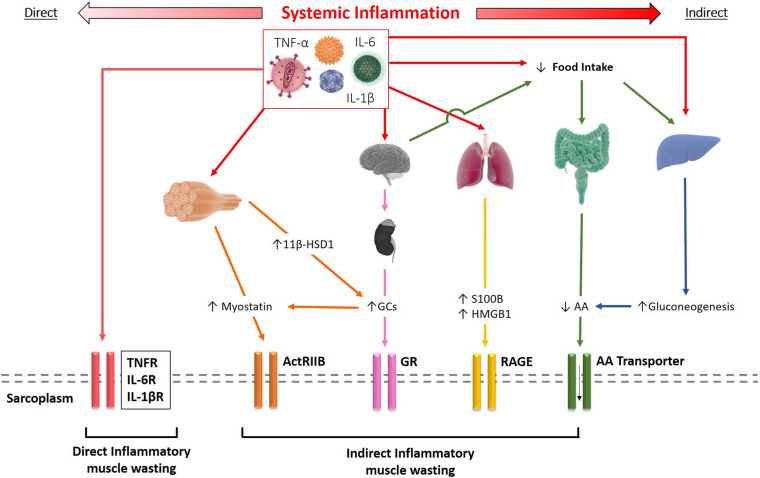
Schematic representation of the direct and indirect effects of systemic inflammation resulting in muscle wasting. Inflammatory cytokines such as TNF-α and IL-1β can bind to their receptors on the sarcolemma driving skeletal muscle wasting directly. Alternatively, cytokines may elicit their effects indirectly through several mechanisms, including increased myostatin and glucocorticoid signaling, release of S100B and HMGB1 by inflamed tissue, or reduced amino acid (AA) availability.

In addition to the direct effects inflammatory cytokines induced by receptor-mediated activation of signaling pathways in skeletal muscle, cytokines also cause dysregulation of other tissue and organ systems which indirectly contribute to muscle wasting and cachexia development. As such, dysregulation of the hypothalamus-pituitary-adrenal (HPA) axis and adrenocorticoids, anorexia and malnutrition, changes in adipocyte behavior, and hepatic metabolism have shown to impact cachexia progression (see overview in Figure 2).

### Involvement of the HPA Axis and Corticosteroid Synthesis in Cachexia

Glucocorticoids such as cortisol are endogenous pleotropic hormones that play an essential role in glucose mobilization and energy metabolism, as well as having potent anti-inflammatory immune modulatory actions (Barnes, 1998). In the 1950s, Kendall, Reichstein and Hench received a Nobel prize for their work in the isolation and application of the GCs in the treatment of chronic inflammatory diseases such as RA. Unfortunately, the clinical efficacy of endogenous and synthetically derived GCs was tempered by severe metabolic side effects, including osteoporosis, truncal obesity, and muscle wasting. The mechanisms underpinning GC induced muscle wasting are compound, with evidence of reduced anabolic IGF-1 signaling and increased anti-anabolic myostatin production, resulting in a reduction in the mTOR signaling pathway, as well as induction of catabolic UPS and autophagy mediated muscle protein degradation, secondary to increased FOXO1 signaling (Gayan-Ramirez et al., 1999; Imae et al., 2003; Fenton et al., 2019), and decreased myogenesis (Pansters et al., 2013).

Due to their muscle atrophy-inducing actions, considerable interest exists regarding possible dysregulation of endogenous steroid synthesis in conditions such as chronic inflammation and cancer cachexia. In particular, the HPA axis is a central regulatory target activated in response to systemic inflammation and stress that has been widely investigated (Ulrich-Lai et al., 2006; Engeland et al., 2016). This critical homeostatic regulatory pathway mediates the synthesis and release of the endogenous GC hormone, cortisol, from the zona fasciculata of the adrenal cortex (Silverman and Sternberg, 2012). Classically, the HPA axis is under central circadian regulation by the hypothalamus, where it drives a pulsatile diurnal secretion of cortisol through the release of corticotropin-releasing hormone (CRH) from the paraventricular nucleus. This in turn results in the systemic release of adrenocorticotropin (ACTH) from the anterior pituitary, which binds to the MC2R receptor in the adrenal cortex to initiate adrenal cortisol synthesis and release (Silverman and Sternberg, 2012; Ruggiero and Lalli, 2016). During inflammation, pro-inflammatory factors such as TNF-α, IL-1β, and IL-6 can act at all levels of the HPA axis to increase CRH, ACTH from the hypothalamus and pituitary, and cortisol synthesis and release from the adrenals (Mastorakos et al., 1993; Nolten et al., 1993; Curti et al., 1996). Conversely, negative regulation of this pathway is achieved through the direct suppression of CRF and ACTH by cortisol.

In particular, focus has fallen upon the concept that either inflammatory cytokines, or disease treatments (such as chemotherapy in cancer) (Braun et al., 2014), cause dysregulation of central hypothalamic/pituitary negative feedback resulting in the over activation of the HPA axis in cachexia (Braun et al., 2011), leading to circulating steroid excess and GC induced muscle wasting as well as loss of adipose tissue (Crofford, 2002; Russell and Tisdale, 2005). In addition, the mixed immunomodulatory actions that circulating GCs may mediate in cancer immunity and in suppression of pro-inflammatory cytokines may also influence disease progression and cachexia. However, studies exploring the dysregulation of the HPA axis specifically in cachexia have yielded mixed results. In mice, several studies report increased activity of the HPA axis in models of cancer cachexia and COPD, coinciding with weight loss (Russell and Tisdale, 2005; Crespigio et al., 2016; Flint et al., 2016; de Theije et al., 2018). de Theije et al. (2018) showed that hypoxia-induced muscle wasting in a murine model of COPD was in turn dependant on GC signaling with GC receptor/KO mice being partly protected from muscle wasting. Several studies in cancer patients (including colorectal, prostate, and breast) report dysregulation of the HPA axis with increased levels of serum cortisol (Strassmann et al., 1992; Soygur et al., 2007; Fearon et al., 2013; Flint et al., 2016). In several of these instances, increases in serum GCs were linked with elevated levels of the pro-inflammatory cytokine IL-6, suggesting this may be a key mediator of increased HPA axis activity. However, whether these changes regulated cachexia in addition to influencing tumor immunity was not fully elucidated (Flint et al., 2016). In addition, muscle GC signaling was found to be required for cancer-induced cachexia (Braun et al., 2013), and muscle atrophy in response to inflammation-evoking cytotoxic chemotherapy was shown to depend on intact GC signaling in skeletal muscle (Braun et al., 2014). Similarly, muscle-specific deletion of GR prevented endotoxin-induced muscle atrophy (Braun et al., 2013). In line, LPS injections in rats induced an inflammatory response, body weight loss, and muscle wasting including upregulation of FoxO and other atrogenes (Schakman et al., 2012). In contrast to TNF-α and NF−κB inhibitors, only inhibition of the GC receptor using RU-486 blunted LPS-induced atrogene expression in this model, highlighting the importance increased GC signaling in inflammation-associated muscle wasting (Schakman et al., 2012). Consequently, the true nature of HPA axis and steroid dysregulation in cachexia, while of significant interest, have proven hard to fully elucidate and show significant disease specific variation.

### A Role for Pre-receptor Steroid Metabolism in Glucocorticoid-Induced Muscle Atrophy

While the systemic regulation of circulating endogenous GC levels is determined through the HPA axis, peripheral exposure to GCs is mediated through their tissue specific pre-receptor metabolism. This is primarily mediated by the 11beta-hydroxysteroid dehydrogenase (11β-HSD) enzymes types 1 and 2 (Hardy et al., 2014). Here, 11β-HSD1 primarily mediates the peripheral conversion of the inactive GC precursor cortisone, to its active counterpart cortisol (11-DHC to corticosterone in rodents) within target tissues, where it greatly amplifies local GC signaling. In contrast, 11β-HSD2 solely inactivates GCs, converting active cortisol to cortisone (corticosterone to 11-DHC in rodents) blocking local GC signaling. 11β-HSD1 shows a diverse pattern of expression across a wide array of tissues including liver, fat, muscle, bone, and in immune cells (Tomlinson et al., 2002; Hardy et al., 2008, 2016). In contrast, 11β-HSD2 expression appears to be limited to tissues such as the kidney where it protects against inappropriate activation of the mineralocorticoid receptor by GCs (Quinkler et al., 2005). Renewed interest in the roles of 11β-HSD1 in inflammatory muscle wasting and cachexia have been fueled by observations that its expression and GC activation are potently upregulated in peripheral tissues such as muscle in response to pro-inflammatory factors such as IL-1β and TNF-α (Ahasan et al., 2012; Hardy et al., 2016). These studies raised the possibility that under conditions of chronic inflammation, local amplification of GC signaling by 11β-HSD1 may represent a critical component in mediating inflammatory muscle wasting. This concept was lent further credit following a seminal study by Morgan et al. (2014) demonstrating that the systemic transgenic deletion of 11β-HSD1 in murine models of GC excess completely abrogated GC induced muscle wasting. However, the only study to examine the role of inflammatory 11β-HSD1 in muscle revealed a complex interplay between the anti-inflammatory actions of GCs versus their anti-anabolic catabolic actions (Hardy et al., 2016). Here, while the transgenic deletion of 11β-HSD1 in murine models of inflammatory polyarthritis resulted in reduced GC signaling in muscle, the exacerbation of muscle inflammation drove a more florid muscle wasting phenotype. Consequently, the role of 11β-HSD1 in other forms of muscle wasting and cachexia requires further investigation.

### Inflammation-Driven Anorexia and Muscle Wasting in Cachexia

Cachexia development is profoundly impacted by the accompaniment of anorexia, categorized by reduced appetite and nutritional deficit which ultimately leads to catabolism of lean body and adipose tissue (Bosaeus et al., 2002). Anorexia–cachexia is distinct from starvation, where skeletal muscle loss is less apparent compared to adipose tissue. Adipose tissue is a reservoir for energy, and therefore in times of starvation or reduced energy intake, catabolism of adipose tissue allows the release of energy which is then used in processes that maintain skeletal muscle mass, however, in cachectic patients both muscle and fat tissue are catabolized as energy sources. In addition, nutritional interventions alone are unable to reverse or alleviate this catabolic phenotype (Moley et al., 1987; Thomas, 2002; von Haehling and Anker, 2010). Although the pathogenesis of anorexia–cachexia is multifactorial, inflammatory cytokines have been shown to be implicated in the development of anorexia in cachectic patients through an amalgamation of mechanisms (Laviano et al., 2003). In some disease states, such as cancer, tumor burden has been implicated in driving anorexia–cachexia through dysphagia or dysregulation of gastro-intestinal function, ultimately leading to reduced food intake and nutritional deficit (Ezeoke and Morley, 2015).

Rats receiving a single dose of human TNF-α resulted in increased muscle proteolysis and anorexia (Bodnar et al., 1989; Flores et al., 1989), while tumor bearing rats receiving TNF-α inhibitors had markedly improved nutritional intake and body weights (Torelli et al., 1999). These findings suggest TNF-α indeed plays a pivotal role in inducing anorexia, although its full contribution the development of cachexia is yet to be elucidated. Plata-Salamán et al. (1988) demonstrated TNF-α administration in rodents suppressed food intake in a dose-dependent manner, through the cytokine directly acting on glucose-sensitive neurons in the central nervous system (CNS) to suppress appetite. Lung cancer patients exhibiting anorexia showed reduced hypothalamic activity compared to non-anorexic patients; however, circulating levels of pro-inflammatory cytokines such as TNF-α, IL-6, and IL-1 were not significantly different between groups (Molfino et al., 2017). Another cytokine implicated in anorexia–cachexia is IL-1, with several studies observing its effects on food intake. These anorexia-inducing effects of IL-1 have been illustrated in several *in vivo* studies, with both peripheral and central administration decreasing food intake in rodents (Kent et al., 1994). Furthermore, Layé et al. (2000) previously showed increases in IL-1 in the hypothalamus in rodents upon LPS administration and reduced food intake, and IL-1 antagonists preventing LPS-induced anorexia. However, many of these *in vivo* studies also show development for tolerance to cytokines, and therefore the interpretation of results is often debated (Mrosovsky et al., 1989). Another study highlights the importance of central regulation of appetite in response to inflammation, with GLP-1 receptor antagonist mitigating anorexia induced by LPS in rats (Grill et al., 2004).

Peripheral hormones that directly affect nutrition status through central actions controlling appetite have also been shown to play an important role in anorexia–cachexia. Ghrelin, a peptide released in the gut shown to stimulate appetite, is decreased in response to acute inflammation (Basa et al., 2003; Otero et al., 2004). In contrast, chronic inflammation in animal models and cachectic patients present with increased ghrelin levels (Nagaya et al., 2001a; Shimizu et al., 2003; Dixit et al., 2004), possibly a compensatory effect of ghrelin resistance in cachectic and catabolic states (Li et al., 2004). Mechanisms underpinning ghrelin’s role in anorexia–cachexia have not yet been established; however, experimental models of cachexia have demonstrated ghrelin administration suppressed weight loss and alleviated skeletal muscle wasting through increased food intake (Nagaya et al., 2001b; Hanada et al., 2003). Another cytokine shown to be of importance in the role of developing cachexia–anorexia is leptin, which is released from adipocytes and signals to the hypothalamus to regulate nutritional intake as a satiety cue. Leptin has been shown to be increased in rodents and humans exhibiting cachexia in many disease states, such as CHF (Cheung et al., 2005). Therefore, the role of leptin in anorexia–cachexia is not well established and is often speculated that the alterations in leptin levels may be in response to malnutrition and reduced fat mass rather than a consequence of elevated inflammatory cytokines.

While these data collectively support a role for pro-inflammatory cytokines in the development of anorexia, due to cachexia’s multifactorial phenotype, it is difficult to underpin the mechanisms in which cytokines may drive anorexia–cachexia. However, a plethora of research indicates that energy deficits in combination with reduced hypothalamic response may play a pivotal role in anorexia–cachexia (Ramos et al., 2004).

### Role of Inflammation-Induced Alterations in Adipocyte and Hepatic Metabolism in Cachexia

There is increasing evidence to show preserving adipose tissue in cachexia can improve mortality and quality of life (Murphy et al., 2010). Lipolysis stimulation during cachexia can be induced by anorexia; however, there is also evidence for inflammation-induced lipolysis. Reduction in food intake or starvation induces lipolysis to release energy stores; however, as lipid stores are depleted, other catabolism of tissues will ensue to provide sufficient energy, of which a main energy source is amino acids derived from skeletal muscle proteolysis (Finn and Dice, 2006). As discussed previously, inflammation can induce anorexia–cachexia, reducing food and energy intake, which ultimately leads to the reduction of fat mass and loss of white adipose tissue (WAT) through lipolysis. Cachexia is also associated with loss of skeletal muscle and WAT through increased energy expenditure (Bosaeus et al., 2001), and related to increased inflammation in pancreatic cancer patients presenting with elevated resting energy expenditure in addition to increased CRP levels (Falconer et al., 1994). Patients with cancer cachexia were shown to have increased levels of circulating IL-6 and enhanced lipolysis compared to weight-stable cancer patients, which was not attributed to enhanced locally expressed IL-6 levels, implicating not the inflammatory infiltrate but other triggers for adipose tissue wasting (Rydén et al., 2008). One mechanism that has been described to contribute to this increased energy expenditure is the remodeling of WAT into brown adipocytes, which has been suggested to occur prior to skeletal muscle wasting in cancer-cachexia (Petruzzelli et al., 2014). This increase in brown adipose tissue increases thermogenesis in these patients (Lee et al., 2010), which ultimately leads to an increased requirement for energy, and thus increased energy expenditure. Interestingly, IL-6 has been implemented in increasing uncoupling protein 1 (UCP1) expression, a protein found in brown adipose tissue that increase thermogenesis (Li et al., 2002). In addition, mice with syngeneic grafts of C26 cells lacking IL-6 showed protection against weight loss and reduced UCP1 expression compared to mice with active IL-6 C26 cells, thus highlighting the importance of this cytokine in WAT browning, and therefore increased energy expenditure in cachexia (Petruzzelli et al., 2014). However, the exact role of WAT browning in skeletal muscle loss in cachexia requires further investigation.

Although various studies have shown the effects of inflammatory cytokines on adipose tissue and skeletal muscle, only few address these in the context of cachexia. Mice s.c. injected with LLC or B16 melanoma cells showed cachexia development, with reduced body weights, WAT loss, muscle wasting, and increased serum TNF-α and IL-6 levels (Das et al., 2011). Inhibition of lipolysis through genetic deletion of adipose triglyceride lipase (Atgl), a mediator of lipolysis, in tumor-bearing animals showed protection against cachexia development, with reduced WAT and skeletal muscle loss. However, TNF-α and IL-6 levels remained increased in the serum of these animals, highlighting a possible indirect action of these cytokines in driving cachexia and adipose tissue loss through lipolytic mechanisms (Das et al., 2011). Importantly, this study also emphasizes the importance of crosstalk between adipose tissue and skeletal muscle, as inhibition of lipolysis resulted in reduced skeletal muscle wasting, therefore suggesting that altered free fatty acid or adipokine release may play a role in skeletal muscle wasting. Adipose tissue secretes adipokines, such as leptin, with endocrine functions including satiety and whole-body metabolism (Galic et al., 2010). Several inflammatory cytokines such as IL-6, TNF-α, and IL-1β are adipokines as well as myokines, and have been implicated in reciprocal control of adipose and muscle mass (Muñoz-Cánoves et al., 2013; Daas et al., 2019) and metabolism (Piya et al., 2013). Further research, however, is required to disentangle the role of inflammation in adipose and muscle reciprocal effects in the context of cachexia.

Although the liver is a central regulator of metabolism, there is relatively little research examining a role of the liver in the association between inflammation and cachexia, which is surprisingly considering the liver is the major site for muscle proteolysis-derived amino acids for utilization in gluconeogenesis and acute-phase protein synthesis, such as CRP (Argilés et al., 2001), and elevated CRP levels are the most frequently applied additional criteria to assess cachexia (Fearon et al., 2006). Indeed, profound hepatic alterations are observed prior to and during the progression of cancer cachexia, including alterations in fat metabolism, collagen deposition, and fibrosis (Rosa-Caldwell et al., 2020). In line, despite not evaluating liver-anatomical changes, alterations in liver metabolism in inflammation associated cachexia have been reported. Apc^*min/+*^ mice with severe cachexia were shown to have increased levels of acute phase protein haptoglobin, revealing hepatic alterations in inflammation-associated cancer cachexia (Narsale et al., 2015). In a model of pancreatic cancer cachexia, inhibition of proliferator-activated receptor-alpha (PPAR-α) through IL-6 resulted in hypoketonemia and subsequent activation of the HPA axis, ultimately leading to increased GC release and enhanced muscle proteolysis (Flint et al., 2016). In addition, Goncalves et al. showed adult *Kras^*G*12*D/+*^;Lkbl^*f/f*^* (KL) mice with lung cancer and cachexia presented with increased IL-6 levels, increased gluconeogenesis in the liver, reduced hepatic fatty acid oxidation, and hypoketonemia. Skeletal muscle atrogenes MuRF1 and Atrogin-1 were upregulated and also noted a decrease in type II fiber CSA (Goncalves et al., 2018). PPAR-α inhibitor fenofibrate restored hepatic ketogenesis, which in turn reduced the requirement for the liver to use gluconeogenesis and alleviate the need for type II skeletal muscle degradation for amino acids (Goncalves et al., 2018). These results therefore show the indirect effects of both liver metabolism and GCs on skeletal muscle wasting in cachexia.

### Myokines as a Nexus and Opportunity in Modulating Inflammation-Associated Cachexia

Apart from the participation of other tissues as an intermediary step between inflammation and induction of muscle wasting, a role for autocrine, paracrine, and even endocrine acting signals derived from skeletal muscle in cachexia is emerging. These concern the “myokines,” e.g., cytokines, growth factors, and other peptide-based molecules released from skeletal muscle (Pedersen et al., 2003). In the context of muscle mass regulation, Mstn is a well-characterized myokine for its muscle growth-inhibitory actions. Increased expression (Kim et al., 2018) and signaling (Zhang et al., 2013) of Mstn in skeletal muscle may constitute an autocrine mechanism of Mstn-dependent muscle wasting in response to inflammatory cues. In line with this notion, blockade of the ActRIIB receptor to inhibit Mstn signaling prevented cachexia in C26 tumor bearing mice, without affecting increased circulating levels of IL-6, TNF-α, and IL-1β (Zhou et al., 2010). Interestingly, increases in muscle Mstn expression and secretion may also contribute to muscle wasting in an endocrine route in case of RA, as Mstn has been implied in inflammatory bone destruction, aggravating RA-associated muscle loss (Fennen et al., 2016). In C26 tumor-bearing BALB/c mice, involvement of myokines was further indicated as increased muscle IL-6, IL-6R, and myostatin expression accompanied muscle wasting in these mice (Lee et al., 2019). Recent work has revealed GDF15 as a myokine, which is expressed at low levels during homeostasis, but can be induced by muscle contraction (Laurens et al., 2020), or metabolic stress (Ost et al., 2020) or increased GDF11 levels (Jones et al., 2018) in skeletal muscle. GDF15 circulating levels correlate inversely with skeletal muscle mass in COPD (Patel et al., 2016), and increasing GDF15 levels are sufficient to induce dramatic weight loss (Johnen et al., 2007). As thus far no evidence supports the expression of the GDF15 receptor, GFRAL, a co-receptor of the Ret tyrosine kinase, in skeletal muscle, this suggests endocrine effects of GDF15 when secreted as a myokine. As such, muscle derived GDF15 was reported to stimulate lipolysis in adipocytes (Laurens et al., 2020), which in the context of cachexia could contribute to adipose tissue depletion. Importantly, elevation of GDF15 suppresses appetite via activation of hypothalamic neurons (Johnen et al., 2007), and the cachexia-inducing properties GDF15 are thought to be a result of anorexia (Johnen et al., 2007). Although induction of GDF15 expression by TNF-α and NF-κB regulation has been shown for other cell types (Ratnam et al., 2017), it remains to be explored whether GDF15 expression increases in skeletal muscle in response to inflammatory cytokines.

In contrast, other myokines including IL-15 (Tamura et al., 2011) and musculin (Nishizawa et al., 2004) have been attributed anabolic effects or anti-catabolic effects on skeletal muscle, at least in part mediated through autocrine mechanisms. In addition, IL-6, when secreted by skeletal muscle in response to stimuli such as exercise, exerts endocrine effects such as lipid oxidation (van Hall et al., 2003), which contribute to organismal homeostasis. Interestingly, in tumor-bearing mice, exercise was found to attenuate tumor growth, which correlated with increased IL-6 levels post-exercise, and systemic IL-6 blocking experiments revealed IL-6 may actually contribute to hindering tumor growth (Pedersen et al., 2016). Moreover, exercise-induced increases in IL−6 contribute to an anti-inflammatory systemic environment, by increasing the production of the anti-inflammatory cytokines, IL−1β receptor antagonist (IL−1ra), and IL−10 (Steensberg et al., 2003). A recent murine study suggested that IL-6 may induce either pro- or anti-inflammatory actions depending on cell source (Han et al., 2020), potentially explaining the beneficial, suppressive effects on tumor growth and immunomodulatory actions of muscle derived IL-6. In addition, C2C12 differentiating myoblasts showed increased IL-6 levels during differentiation in combination with increased STAT3 phosphorylation. Blockade of IL-6 independently showed reduced differentiation of myotubes, highlighting the crucial role IL-6 has in differentiating myotubes (Hoene et al., 2012). As such, these studies may reflect an endocrine cachexia-modulating potential of myokines.

## Conclusion and Future Perspectives

Despite the overwhelming preclinical evidence to imply inflammation as both sufficient and required in disease-associated cachexia, this has not translated into unambiguous success of cytokine-depleting therapeutic agents to reverse cachexia in patients with cancer or chronic disease. This may reflect the complex interactions within an inflammatory response, rendering a therapy based on inhibition of a single cytokine therapy insufficient. Combined blocking approaches (Truyens et al., 1995), or downstream inhibition of molecules at which inflammatory cues convergence, like NF-κB (Miao et al., 2017) or STAT3 (Ahasan et al., 2012) have shown effective in experimental models and warrant further exploration for pharmacological modulation. In addition, the timing of anti-inflammatory treatment may be of key importance. Much of the evidence in the experimental models indicates inflammation precedes a cachectic phenotype, and anti-inflammatory interventions successfully modulating cachexia in preclinical studies are without exception started prior to initiation of cachexia development. Consequently, for anti-inflammatory agents to be effective in a clinical setting, this may require interventions to start in patients at risk for cachexia, i.e., “pre-cachectic,” for subsequent evaluation of their ability to prevent or delay onset of cachexia. Bearing time in mind as an important determinant of the efficacy anti-inflammatory modulation, its downstream signaling should be further considered. Feed-forward signals that transform inflammatory cues of systemic origin into an autocrine, muscle atrophy-promoting signal, have been reported for Mstn (Zhang et al., 2013) and Activin-A (Trendelenburg et al., 2012). Receptor blocking agents for these ligands are available, and the first clinical trials have yielded promising results in terms of safety and efficacy in COPD patients with low muscularity (Polkey et al., 2019). These ActRIIB inhibitors are continuously refined (MacDonald et al., 2012), and further improvement in their efficacy to halt or even reverse cachexia progression is anticipated when provided as an integral part of a multimodal therapy, i.e., combined with appropriate nutritional support and tailored exercise programs. Similarly, desensitizing skeletal muscle to the atrophy-inducing effects of GCs, by inhibition of local GC-activation using therapeutic 11β-HSD1 inhibitors that are currently in clinical trials for other applications (Harno and White, 2010), may be a route to explore the potential of blocking the indirect effects by which inflammation contributes to muscle wasting. Finally, skeletal muscle tissue itself may hold the key to counteracting inflammation driven cachexia, as myokines have been attributed very potent immunomodulatory features, which in future research deserve further investigation in their potential to prevent and reverse skeletal muscle wasting in cachexia.

Combined, the extensive efforts to delineate the underlying mechanisms of inflammation-associated cachexia have revealed insights that provide multiple leads to evaluate novel, more selectively targeted therapeutic approaches in this debilitating condition.

## Author Contributions

JW and LK systematically reviewed potentially relevant manuscripts to extract and synthesize the findings into the various sections of the review. RL conceived the focus of the manuscript. RH and RL defined the structure of the review, critically edited the content of the paragraphs, and defined conclusions. All authors contributed to the article and approved the submitted version.

## Conflict of Interest

The authors declare that the research was conducted in the absence of any commercial or financial relationships that could be construed as a potential conflict of interest.
